# Diverse cropping systems lead to higher larval mortality of the cabbage root fly (*Delia radicum*)

**DOI:** 10.1007/s10340-023-01629-1

**Published:** 2023-05-05

**Authors:** Peter N. Karssemeijer, Luuk Croijmans, Karthick Gajendiran, Rieta Gols, Dirk F. van Apeldoorn, Joop J. A. van Loon, Marcel Dicke, Erik H. Poelman

**Affiliations:** 1 Laboratory of Entomology, Wageningen University & Research, P.O. Box 16, 6700 AA Wageningen, the Netherlands; 2 Farming Systems Ecology, Wageningen University & Research, P.O. Box 430, 6700 AK Wageningen, The Netherlands; 3 Field Crops, Wageningen University & Research, Edelhertweg 10, 8200 AK Lelystad, The Netherlands

**Keywords:** Root herbivory, Crop diversification, Aboveground–belowground interactions, Strip cropping, White cabbage (*Brassica oleracea*), Cabbage root fly (*Delia radicum*)

## Abstract

Root herbivores pose a major threat to agricultural crops. They are difficult to control and their damage often goes unnoticed until the larvae reach their most devastating late instar stages. Crop diversification can reduce pest pressure, generally without compromising yield. We studied how different diversified cropping systems affected the oviposition and abundance of the specialist cabbage root fly *Delia radicum*, the most important root herbivore in *Brassica* crops. The cropping systems included a monoculture, pixel cropping, and four variations of strip cropping with varying intra- and interspecific crop diversity, fertilization and spatial configuration. Furthermore, we assessed whether there was a link between *D. radicum* and other macroinvertebrates associated with the same plants. Cabbage root fly oviposition was higher in strip cropping designs compared to the monoculture and was highest in the most diversified strip cropping design. Despite the large number of eggs, there were no consistent differences in the number of larvae and pupae between the cropping systems, indicative of high mortality of *D. radicum* eggs and early instars especially in the strip cropping designs. *D. radicum* larval and pupal abundance positively correlated with soil-dwelling predators and detritivores and negatively correlated with other belowground herbivores. We found no correlations between the presence of aboveground insect herbivores and the number of *D. radicum* on the roots. Our findings indicate that root herbivore presence is determined by a complex interplay of many factors, spatial configuration of host plants, and other organisms residing near the roots.

**Supplementary Information:**

The online version contains supplementary material available at 10.1007/s10340-023-01629-1.

## Introduction

Insect root herbivores are major agricultural pests, causing damage in many crops, including maize, onion, and cabbage (Brown and Gange 1990; Finch 1989; Johnson et al. 2016). Due to their belowground life stages, root herbivores often go unnoticed and can be difficult to control. This is especially true for pest species with larvae that feed within and on the surface of roots or bulbs, such as the larvae of the cabbage root fly *Delia radicum* and the onion fly *D. antiqua* (Hunter 2001). Root herbivores are often controlled by preventive applications of insecticides (Johnson et al. 2016). These insecticides have detrimental effects on many species in the agro-ecosystem, including pollinators, parasitoid wasps, aquatic macroinvertebrates in farmland streams, and insectivorous birds (Calvo–Agudo et al. 2019; Hallmann et al. 2014; Kessler et al. 2015; Schulz and Liess 1999). Moreover, insecticides affect human health (Goldman 2014). To move towards sustainable agriculture, the use of insecticides should be reduced and alternatives are needed. One of these alternatives is to increase diversity in the agroecosystem to enhance ecology-based pest suppression.

Increasing diversity in agroecosystems has great potential to reduce pest outbreaks without compromising yield (Juventia et al. 2021; Mansion–Vaquié et al. 2020; Parker et al. 2016; Tajmiri et al. 2017). Cropping systems can be optimized to maximize benefits by increasing genetic, temporal, and spatial diversity (Ditzler et al. 2021a). Intercropping, i.e. growing multiple crops in close proximity, is a promising practice to reach higher crop diversity. Crop diversification can enhance yield, as plants benefit from factors such as niche differentiation and more efficient resource use (Li et al. 2020; Yu et al. 2015). Two recent meta-analyses showed that intercropping increases yield by about 30% compared to monocultures (Beillouin et al. 2019; Li et al. 2023), although in grains the best-producing sole crop still generally has a slightly higher grain yield than intercropped crops (Li et al. 2023). Intercropping may also reduce pest outbreaks through interference with host-searching behaviour of the pest (Finch and Collier 2000; Mansion-Vaquié et al. 2020), by reducing the concentration of host plants (resource concentration hypothesis) (Root 1973), or by enhancing the abundance, diversity and control efficiency of natural enemies (natural enemies hypothesis) (Khan et al. 1997; Nilsson et al. 2016; Tajmiri et al. 2017; Trenbath 1993). Inclusion of a nectar-providing crop, for instance, can boost the number and longevity of parasitoid wasps that parasitize insect herbivores (Nilsson et al. 2016), although floral resources might also positively benefit oviposition and longevity of herbivorous insects of which the adults are nectarivorous (Nilsson et al. 2011). Further, crop diversification can lead to increased abundance and diversity of ground-dwelling arthropods, which may prey on eggs and larvae of root herbivores (Booij et al. 1997; Finch 1989).

Various forms of intercropping are distinguished based on the spatial configuration of plants on the field, such as strip cropping and pixel cropping. In strip cropping, two or more crops are grown adjacent to one another in alternating, long and relatively narrow strips (Juventia et al. 2022). In pixel cropping, crops are planted in a patchwork of squares, with a surface area of below 2 square metres per square. Strip cropping of just two crops can already reduce pest pressure and enhance biodiversity (Li et al. 2019; Ma et al. 2007; Tajmiri et al. 2017). Further diversification by including more crops in the strip cropping scheme (Parker et al. 2016), mixing of cultivars (Koricheva and Hayes 2018; Wetzel et al. 2018), or reducing fertilizer inputs via additional nitrogen-fixing plants (Ditzler et al. 2021b) could benefit the agro-ecosystem even more.

We studied the interactions between white cabbage, *Brassica oleracea* L. var. Capitata (Brassicales: Brassicaceae), and its herbivorous insect community, with a focus on the cabbage root fly *D. radicum* L. (Diptera: Anthomyiidae) in an agricultural setting. Cabbage root flies are specialized on plants in the Brassicaceae family and are an important pest of cabbage in temperate regions (Finch 1989). Females select their host plants using visual and chemical cues gathered from aboveground tissues (Kergunteuil et al. 2015; Roessingh and Städler 1990; Zohren 1968). Eggs are laid in the soil just next to the plant stem, and upon hatching the larvae make their way to the tap root and feed on and within it. After three to four weeks, larvae pupate in the soil surrounding the plant (Collier and Finch 1985). In temperate regions such as the UK, there are two to three generations of cabbage root fly per season, but this is likely to increase as a result of climate change (Collier et al. 1991). A variety of insect natural enemies are responsible for top-down control of *D. radicum*: the larval parasitoid *Trybliographa rapae* W. (Hymenoptera: Figitidae), the pupal parasitoids *Aleochara bipustulata* L. and *Aleochara bilineata* G. (Coleoptera: Staphylinidae) and predatory ground-dwelling beetles (e.g. carabids or staphylinids) prey on the eggs (Finch 1989; Han et al. 2022; Mesmin et al. 2022). We also examined the interactions between *D. radicum* and the community of aboveground cabbage herbivores. *D. radicum* performance and oviposition are known to be affected by the presence of other herbivores, which might be a source of bottom-up control via changes in plant quality and attractiveness (Karssemeijer et al. 2022a, 2020, 2022b).

Here, we assessed how a range of increasingly diverse intercropping practices affected the oviposition and infestation by *D. radicum*. To improve management of *D. radicum* infestation, various intercropping studies have been carried out during the past decades (Björkman et al. 2010; Finch and Collier 2000; Lamy et al. 2017a; Langer 1996; Meyling et al. 2013; Nilsson et al. 2016, 2012; Tukahirwa and Coaker 1982). Most studies have focused on a mixed cropping system with cabbage and a living mulch of clover, a nitrogen-fixing plant, which provides some level of control against *D. radicum* through interference with egg-laying behaviour (Finch and Collier 2000). Here, we used different cropping systems, in which we tested how *D. radicum* was affected by increasing spatial crop diversity, enhancing intraspecific variation via cultivar mixing, and the addition of nectar sources. This study is part of a long-term systems experiment in which complete cropping systems were compared, rather than a factorial approach in which specific characteristics are the focus (Drinkwater 2002). First, we monitored both *D. radicum* oviposition and larval/pupal abundance in and around the roots. We expected to find fewer *D. radicum* eggs, larvae and pupae in more diverse cropping systems, due to a reduced concentration of host–plant resources and enhanced predation. Next, we assessed whether the communities of above- and belowground herbivores correlated with *D. radicum* egg, larval and pupal abundance. Previous greenhouse studies indicated that leaf-chewing herbivores negatively affected *D. radicum* performance, whereas oviposition was stimulated on induced plants (Karssemeijer et al. 2020, 2022b; Soler et al. 2007). Therefore, we expect that the abundance of leaf-chewing herbivores is positively correlated with *D. radicum* oviposition and negatively correlated with the abundance of *D. radicum* larvae and pupae. Lastly, we examined the community of natural enemies of *D. radicum* by rearing collected pupae from the field and by deploying pitfall traps. We expected the abundance of both generalist and specialist natural enemies to be higher in more diverse cropping systems.

## Materials and methods

### Field setup

**Fig. 1:**
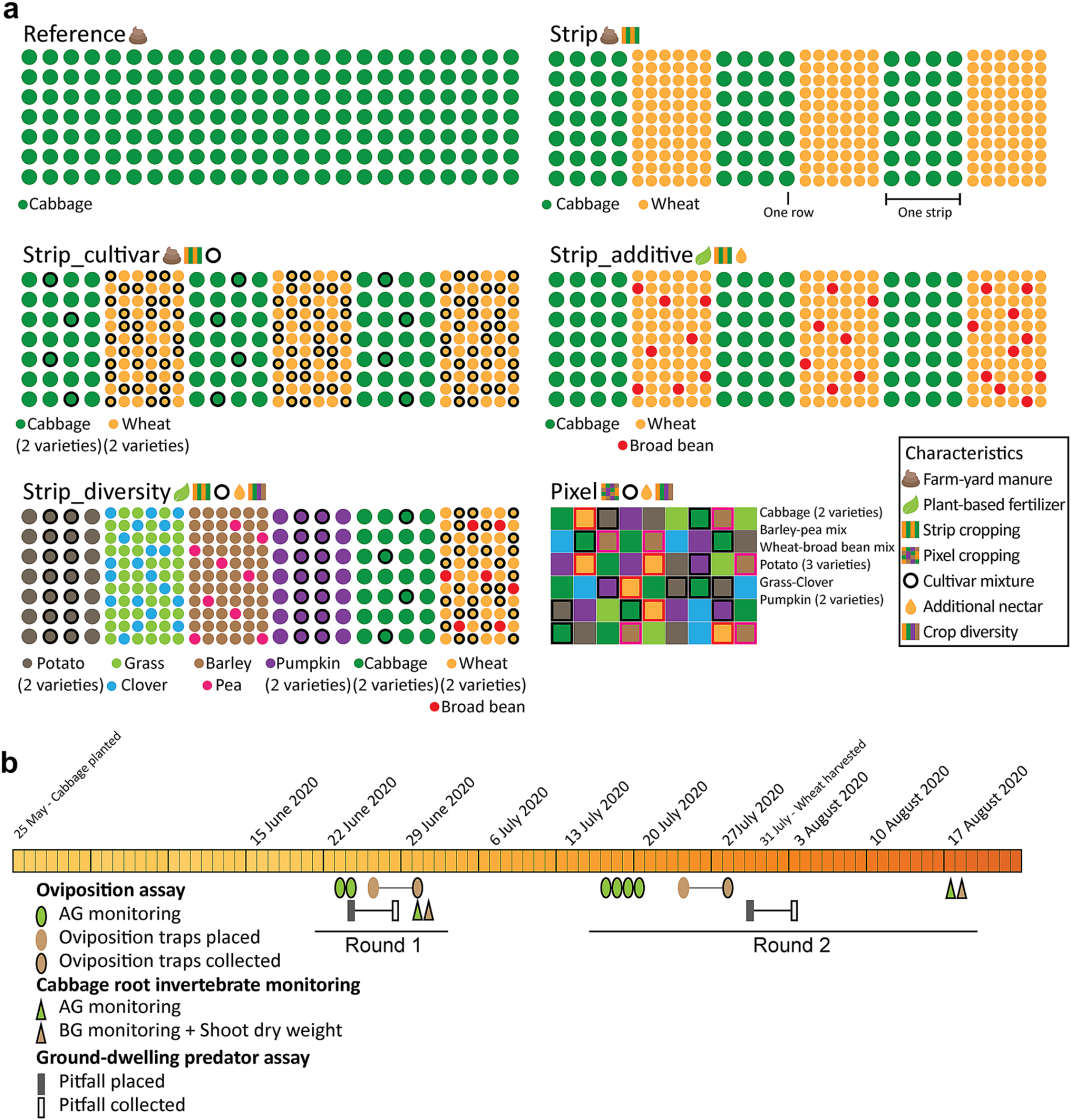
Experimental setup. **a** Illustrations of the different cropping systems (treatments). Each panel represents one replicate in the field, in which measurements were taken in the central cabbage strips. Circles represent a plant, and black outlines represent a second crop cultivar. Icons behind treatment names indicate cropping system characteristics: type of fertilization, strip cropping, cultivar mixture, addition of a nectar source, increased crop diversity (i.e. six crops), pixel cropping. **b** Timeline of the experiments throughout the field season of 2020. AG: aboveground, BG: belowground

This study was carried out in the summer of 2020 as part of a large long-term strip-cropping trial at Droevendaal Experimental Farm in Wageningen, the Netherlands (Juventia et al. 2021). At the field site, white cabbage (*Brassica oleracea* var. Capitata), wheat (*Triticum aestivum* L.), pumpkin (*Cucurbita maxima* L.), potato (*Solanum tuberosum* L.), barley (*Hordeum vulgare* L.), and grass (*Lolium multiflorum* L.) were grown as main crops (Fig. 1a). The experimental design consisted of six cropping designs (Fig. 1a): (1) Reference, i.e. cabbage monoculture of 51 by 45 m (cv. Rivera), (2) Strip, i.e. alternating strips of cabbage (cv. Rivera) and wheat (cv. Lennox), (3) Strip_cultivar; alternating strips of two cultivars of cabbage (cv. Rivera and Christmas Drumhead) and two cultivars of wheat (cv. Lennox and Lavett), (4) Strip_additive, i.e. alternating strips of cabbage (alone) and a combination of wheat with broad bean (*Vicia faba* L., cv. Pyramid) to add floral and extrafloral nectar to the system for natural enemies of herbivores, (5) Strip_diversity; composite of strips of all six crops with two cultivars per crop and a nectar source in the Poaceae crops (wheat with broad bean, barley with pea, grass with clover), (6) Pixel, adjacent 50 × 50 cm plots with each containing one of the six crops with two varieties per crop and a nectar source in the poaceous crops, in a random design. The same crops and additional nectar sources were planted in the Strip_diversity and Pixel-cropping designs, but with a different spatial configuration.

The setup was an incomplete block design on four adjacent fields. In one field, the reference monoculture (51 by 45 m in size) and a replicate of the strip cropping design were planted next to each other. The other three fields contained replicates of all strip cropping designs (Strip, Strip_additive, Strip_cultivar, Strip_diversity). Every replication of the Strip, Strip_additive and Strip_cultivar cropping designs consisted of three strips per crop, of which the central two strips of cabbage were included in the measurements to reduce interference between cropping designs. In the Strip_diversity cropping design, a single strip of each crop was planted per replication. In all strip cropping designs, each strip was 3 m wide and 42 or 54 m in length. Two replicates of the Pixel cropping design were planted in the fields, each measuring 12 by 7.5 m consisting of 360 pixels. Planting distance of cabbage was 38 cm within rows and 75 cm between rows, resulting in four rows per strip. In the Strip_cultivar and Strip_diversity cropping designs, the cabbage cultivar “Christmas Drumhead” was planted in the centre two rows of each strip, as every fourth plant (Fig. 1a). We expected this cultivar to attract shoot herbivores and parasitoids (Poelman et al. 2009a; Poelman et al. 2009b), serving as a trap crop and attracting natural enemies into the strip. This second cultivar was also included in the Pixel-cropping design.

Fertilization was carried out two weeks prior to planting and varied between the cropping designs. One of the overarching goals of the field trial in which this study was carried out is to study different cropping systems, including differences in fertilization strategies. The Reference, Strip, and Strip_cultivar cropping designs received farm yard manure (35 t/ha), the Strip_additive and Strip_diversity cropping designs were fertilized using organic plant fertilizer (11–0-5 NPK) in a concentration matched to the manure in terms of nitrogen dosage. The Pixel cropping design should have received a similar fertilization as the Strip_additive and Strip_diversity cropping designs; however, due to unforeseen circumstances, this was withheld. Moreover, there were differences in the precrop between the cropping designs, most notably the precrop for the Strip_additive, Strip_diversity and Pixel cropping designs included red clover (*Trifolium pratense* L.), a nitrogen fixer. The rotation scheme of the long-term field trial ensured that each cropping design was planted on a plot that had a similar cropping design in the previous year, but the six main crops were rotated over a six-year rotation scheme (i.e. a strip containing the Strip_diversity cropping design in one year will be Strip_diversity again in the next year, but with a different main crop). Cabbage plants were planted on the 25th of May and harvested from the 15th of October until the 26th of November (Fig. 1b). The wheat intercrop was sown in the second half of March and harvested on July 31st, meaning that these rows were empty for two weeks before the last measurements were taken.

### Data collection

All data presented in this study relate to the white cabbage crop (cv. Rivera).

#### *Delia radicum* oviposition and shoot herbivore monitoring

We assessed the oviposition by cabbage root flies in different cropping systems using felt traps, a commonly used method (Bligaard et al. 1999; Lamy et al. 2020). Strips (4 × 100 cm) of black felt were wrapped around the stem of cabbage plants and secured using a safety pin. Felt traps were collected five days after placing them and the number of eggs in each trap was counted. Three to seven days prior to placing the felt traps, the community of aboveground herbivores on each plant was assessed. Herbivores present on the plant were directly identified to the species level (except thrips and leaf miners). We measured the maximum plant radius (distance between leaf tips furthest apart) as a non-destructive assay of plant size. Per cropping design, 16 cabbage plants were assessed in each cropping design replicate. The first and last 10 m of each strip were not sampled, to account for field edge effects. For the Strip, Strip_additive, Strip_cultivar and Reference, two strips with each eight plants and in the Strip_diversity one strip with 16 plants were sampled. In the Reference monoculture the distance between replicate strips was largely exceeding the distance between replicates of strip cropping treatments, and therefore considered independent measures. The sampled plants were equally spread throughout the strip, and the order of the rows to be sampled was randomized. In the Pixel cropping design, 16 random cabbage plants were chosen among the cabbages that were not on the edges of the Pixel field.

#### Cabbage root and shoot invertebrate monitoring

To examine how cropping systems affected *D. radicum* larval and pupal abundance, and how these larvae and pupae correlated with the community of invertebrates associated with cabbage plants, we sampled both the above- and belowground invertebrate communities of cabbage plants. For this purpose, we chose eight cabbage plants (two per plant row) per experimental strip in two rounds: 29–30 June and 17–18 August (Fig. 1b). As this was a time-consuming sampling effort, timing was important to assure peak *D. radicum* presence. Therefore, throughout the season, we monitored a small number of cabbage plants in the fields every other week to confirm the presence of *D. radicum* larvae and pupae (Table S1). This pilot data, together with the oviposition data, provided the basis for timing the cabbage root and soil collection when *D. radicum* densities peaked.

First, we assessed the aboveground community of herbivorous insects by visually examining cabbage plants. Here, all herbivores were identified to the order level (thrips), morphospecies (leaf miners) or species level (all other herbivores) and counted.

Secondly, we examined the belowground arthropod community by taking soil samples around the root of the cabbages. For this purpose, we cut the cabbage plant at the base of the stem and we took a soil sample using an auger (20 cm diameter) to a depth of 20 cm with the cabbage tap-root at the centre, one day after the aboveground herbivore monitoring. As such, 16 cabbages were removed from the strips. We expect only minor effects of this removal as the shortest strips already contained about 450 cabbages. Soil samples were placed in plastic bags, secured using a tie-wrap, and stored at 7 °C until further analysis. The Pixel cropping design was not included in this monitoring, as removal of plants would have interfered too much with other measurements in this cropping design.

Within one week after collection and storage, soil samples were thoroughly searched for macroinvertebrates. The cabbage taproots were carefully opened to find *D. radicum* larvae feeding within. When found, these cabbage root fly larvae were placed in small containers containing a small piece of rutabaga (*Brassica napus* var. *napobrassica*) to rear them to pupation. The collected pupae were stored in glass vials in a climate cabinet (20 ± 1 °C) until eclosion to assess parasitism. While we did not identify *Delia spp.* to the species level, a previous study indicates that the community in Northern Europe is dominated by *D. radicum*, and a small fraction of *D. floralis* (Klingen et al. 2000). We therefore assume most specimens found in this study to be *D. radicum*. Empty *D. radicum* puparia collected from the soil samples were also scored.

Lastly, as we expected that plant size might also affect *D. radicum* larval/pupal abundance, we also quantified plant dry biomass. For this purpose, cabbage plant shoots were collected in paper bags and shoot tissue was dried at 70 °C for 2 days and weighed on a “DK-6200-C-M” balance (± 0.1 g).

#### Pitfall trapping

Furthermore, two times during the season, pitfall traps were placed to quantify the abundance of (egg) predators (Fig. 1b). Pitfall traps consisted of a plastic cup (8.5 cm diameter) with a layer of water 3 cm high with odourless dish soap placed in the soil up to the rim, which was covered with a plastic roof (12.5 cm diameter) approximately 2 cm above the soil surface. One pitfall trap was placed in a predetermined random position in the one of the central rows of each strip, and traps were left in the field for 5 days. Macroinvertebrates captured in the pitfall traps were preserved in 70% ethanol and identified. Carabid beetles were identified to the species level (Muilwijk et al. 2015). Only the activity densities of staphylinid and carabid beetles were statistically analysed.

### Statistical analysis

Data were analysed using R (R Core Development Team 2017), with packages vegan (Oksanen et al. 2007), lme4 (Bates et al. 2015), emmeans (Lenth et al. 2018), ggeffects (Lüdecke 2018). Individual plants or pitfall traps were the statistical units. Most of the data were counts, which we analysed using Generalized Linear Mixed Models (GLMM) with a negative binomial distribution. Plant quality measurements, i.e. shoot dry biomass and maximum radius, were analysed with a (G)LMM with a gamma or normal distribution. Field (a factor indicating the four fields on which the experiment was replicated) was included as a random factor. We analysed these response variables both using separate datasets for the two rounds, and for both rounds combined. In the case of continuous explanatory variables, we used the ggpredict function to generate conditional predictions of the correlation between the response variable and the explanatory variables whilst keeping other variables and the random factor constant. All models were validated using the DHARMa package (Hartig 2020), where we tested the residuals of all models. To assess whether the four fields showed similar patterns in oviposition or larval / pupal abundance, we also compared the strip cropping design on all four fields using the same statistical methods as mentioned above (Fig. S1a, c). To check if the reference field responded in line with our models from data of all fields combined, we also compared oviposition and larval / pupal abundance between the monoculture reference and strip cropping design on the reference field specifically (Fig. S1b, d).

We performed a principal component analysis (PCA) with auto-scaling using the Hellinger transformation, on the community of aboveground herbivores (oviposition data) and total macroinvertebrate community (soil sample data) separately for each round. Using redundancy analysis (RDA) in which we added the number of cabbage root fly eggs or larvae and pupae as an explanatory variable, we further established whether cabbage root fly oviposition or abundance was correlated with other members of the plant-associated macroinvertebrate community. Finally, we tested for correlations between cabbage root fly oviposition and abundance and other macroinvertebrates using GLMM. For this analysis, species were grouped into explanatory variables based on feeding site and guild into: aboveground chewers, aboveground phloem-feeders, belowground detritivores, belowground predators, and belowground herbivores. No analyses were performed on parasitism rates, as the number of parasitized larvae / pupae found was too low for meaningful comparisons.

Lastly, we analysed carabid and staphylinid beetle activity densities among the different cropping designs using GLMM with a negative binomial distribution. Here, we used cropping designs, rounds and the interaction between cropping design and round as fixed factors. For these analyses, we used data of both rounds together.

## Results

### *Delia radicum* oviposition and larval / pupal abundance in different cropping systems

**Fig. 2:**
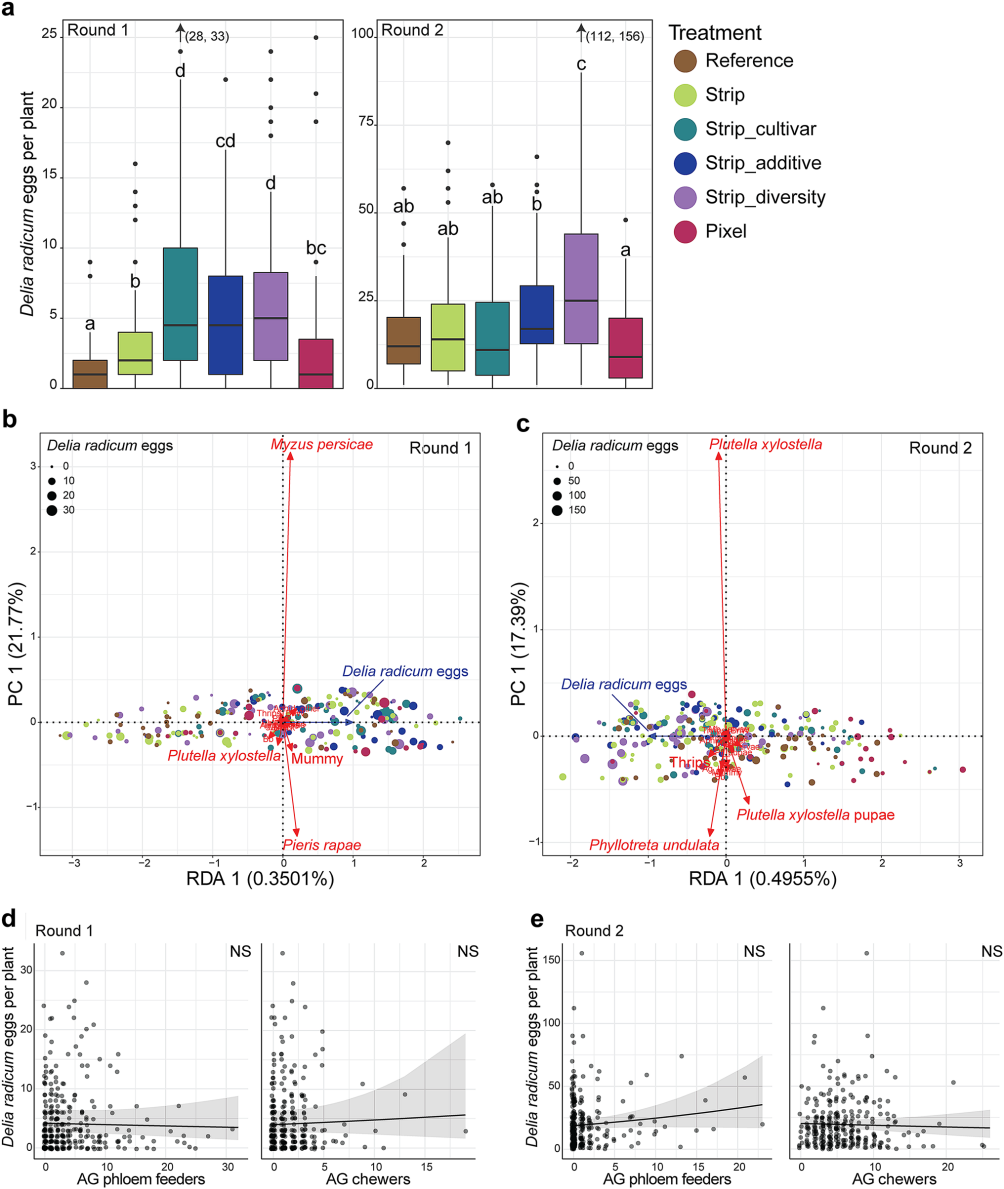
Oviposition of cabbage root fly in a strip cropping farm trial. **a** Effects of strip cropping treatments on the number of *D. radicum* eggs per plant, note that the Y-axis scale differs between the two rounds. Results of pairwise comparisons between treatments are indicated with letters; treatments having no letters in common differ significantly (*P* < 0.05). Arrows indicate outliers outside the plot area, and their values are added between brackets. **b, c** Redundancy analysis (RDA) of the aboveground herbivore community on cabbage plants grown in different cropping designs (treatments) in rounds 1 and 2. Constrained by the number of *D. radicum* eggs collected on these plants. Size of the data points reflects the number of eggs found on felt traps the week after the aboveground herbivore community was assessed. **d, e** Conditional predictions of the relationship between aboveground phloem feeders or chewers and the abundance of *D. radicum* eggs in round 1 and 2; dots are the original data points, the line indicates the predicted values and the grey area depicts 95% confidence interval. AG: aboveground

From the 304 sampled plants we collected 1636 (average 4.08 per plant) and 6249 (average 18.61 per plant) cabbage root fly eggs in the first and second round, respectively. More eggs were collected in round 2 (29 July 2020) compared to round 1 (30 June 2020; Table 1, Fig. 2a). Oviposition by cabbage root flies differed among cropping designs, and how oviposition differed among cropping designs depended on the sampling round (Table 1, Fig. 2a). In the first round, cabbage root fly females deposited the lowest number of eggs per trap on plants in the monoculture (Reference). Compared to the monoculture, traps placed on plants in a pixel cropping design (Pixel) contained slightly more eggs, traps in a strip cropping design with wheat (Strip) contained twice as many eggs, and traps placed on cabbage plants in the three other strip cropping designs (Strip_cultivar, Strip_additive, Strip_diversity) contained roughly four times as many eggs. In the second round, differences were less pronounced than in the first round. Compared to the reference monoculture, traps placed on cabbage plants in strips of six crops (Strip_diversity) contained roughly twice as many eggs. None of the other cropping designs differed from the reference monoculture.

**Table 1:** Model parameters for all univariate models, including: model distribution, random and fixed effects, (conditional) *R*^2^ value, Chi^2^ value and *P* value. To get an idea of field effects, we compared *D. radicum* egg and larval / pupal abundance in the strip-cropping design in each field. To assess whether the reference field showed a response that was in accordance with the models that included all fields, we also analysed the reference field separately, as this field only contained the monoculture reference and the strip-cropping design

Response variable	Distribution	Dataset	Random effects	Fixed effects	R2	Chi-square	*P* value
*D. radicum *Egg abundance	Negative binomial	All data	Field	Cropping system	0.54	33.7	** < 0.001**
				Round		250.0	** < 0.001**
				Cropping system * round		26.8	** < 0.001**
		Only strip cropping design	N/A	Field	0.65	1.87	0.601
				Round		78.3	** < 0.001**
				Field * round		7.94	**0.047**
		Only reference field	N/A	Cropping system	0.77	0.40	0.529
				Round		119.9	** < 0.001**
				Cropping system * round		1.05	0.306
		Round 1	Field	AG phloem feeders	0.19	0.14	0.711
				AG chewers		0.19	0.666
		Round 2	Field	AG phloem feeders	0.06	2.95	0.086
				AG chewers		0.24	0.623
		Round 1	Field	Radius	0.24	5.02	**0.025**
		Round 2	Field	Radius	0.04	0.35	0.553
Plant radius	Gaussian	Round 1	Field	Cropping system	0.34	75.4	** < 0.001**
		Round 2	Field	Cropping system	0.57	146.1	** < 0.001**
*D. radicum *larval and pupal abundance	Negative binomial	All data	Field	Cropping system	0.22	9.85	**0.043**
				Round		5.24	**0.022**
				Cropping system * round		12.9	**0.012**
		Only strip cropping design	N/A	Field	0.29	17.5	** < 0.001**
				Round		1.90	0.169
				Field * round		4.25	0.236
		Only reference field	N/A	Cropping system	0.04	0.00	0.969
				Round		0.25	0.620
				Cropping system * round		3.21	0.073
		Round 1	Field	AG Phloem feeders	0.16	1.24	0.217
				AG Chewers		0.58	0.562
				BG Predators		0.58	0.562
				BG Detritivores		1.11	0.267
				BG Herbivores		-1.21	0.226
		Round 2	Field	AG Phloem feeders	0.33	-0.67	0.500
				AG Chewers		0.04	0.969
				BG Predators		3.15	**0.002**
				BG Detritivores		3.31	** < 0.001**
				BG Herbivores		-2.56	**0.010**
Shoot dry weight	Gaussian	Round 1	Field	Cropping system	0.004	31.3	** < 0.001**
		Round 2	Field	Cropping system	0.30	67.9	** < 0.001**
Carabid beetle activity density	Negative binomial	All data	Field	Cropping system	0.56	23.8	** < 0.001**
				Round		15.1	** < 0.001**
				Cropping system * round		7.13	0.211
Staphylinid beetle activity density	Negative binomial	All data	Field	Cropping system	0.48	3.70	0.593
				Round		22.5	** < 0.001**
				Cropping system * round		1.20	0.945

P values in bold are significant (*α* < 0.05)

In both rounds, plant radius differed between cropping designs (Table 1, Fig. **S2a**). Plants in the reference monoculture grew larger compared to the other cropping designs. At the first measuring point (round 1), plants grown in strips next to wheat, either with or without a second cultivar (Strip, Strip_cultivar), grew larger compared to plants grown in rotated strips with six other crops (Strip_diversity) or strips with broad beans interspaced between wheat (Strip_additive). At the second time point (round 2), plants grown in the pixel-cropping system, grew smaller than any other cropping design. A correlation between the number of cabbage root fly eggs and plant size was found in the first round but not in the second (Table 1, Fig. **S2b**). In the first round, cabbage root fly females oviposited more on larger plants.

**Fig. 3:**
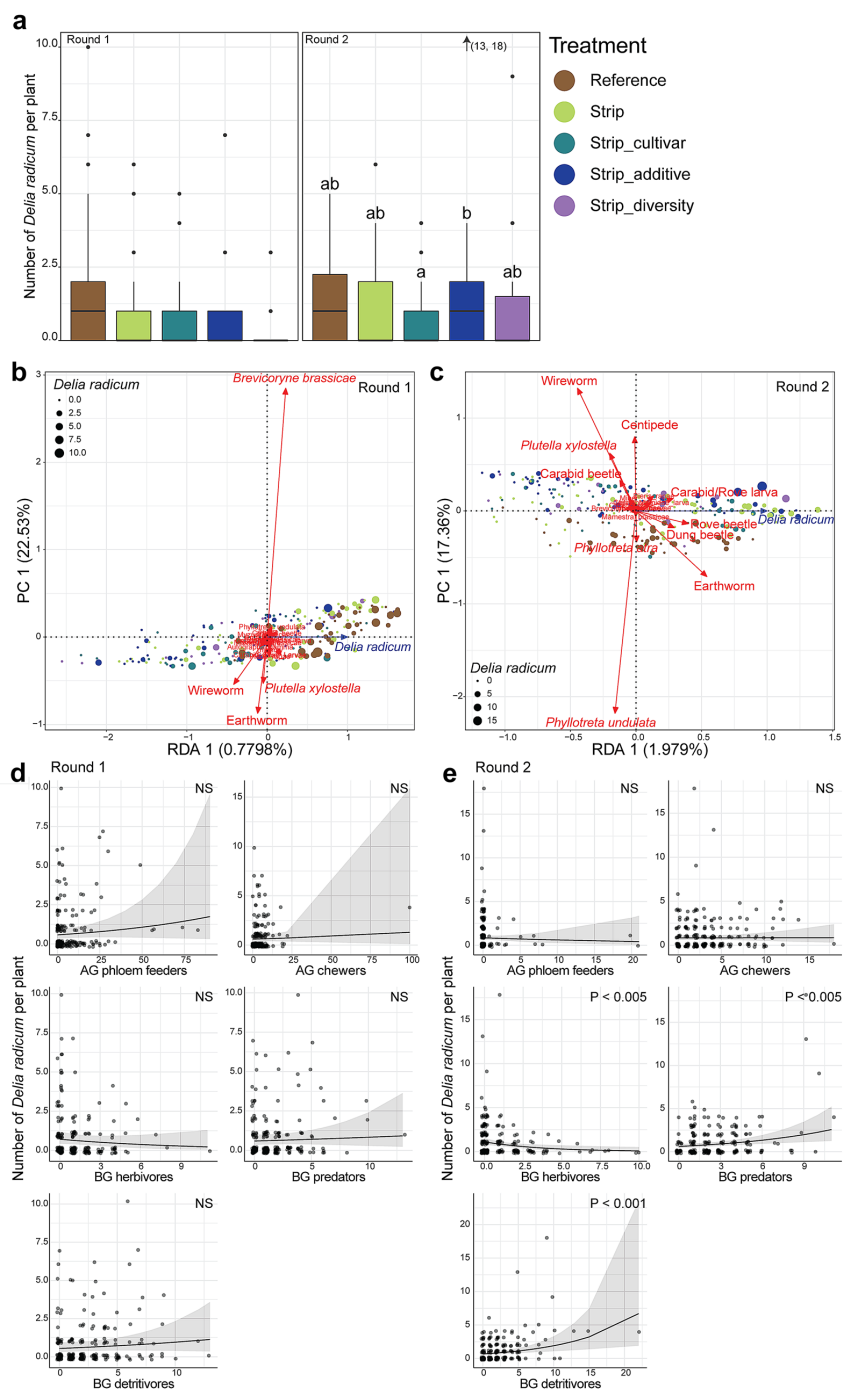
Abundance of *D. radicum* larvae and pupae in a strip cropping field trial. Plants were uprooted and the soil macroinvertebrates was assessed as well as the number of *D. radicum* larvae and pupae in and around the cabbage taproot in a soil core of 20 cm diameter. The day before harvesting, aboveground herbivore communities were assessed. **a** Effects of strip cropping treatments on the number of *D. radicum* larvae and pupae. Results of pairwise comparisons between treatments are indicated with letters; treatments having no letters in common differ significantly (*P* < 0.05). Arrows indicate outliers outside the plot area, and their values are added between brackets. **b, c** Redundancy analysis (RDA) of the above- and belowground macroinvertebrates community on cabbage plants grown in different cropping designs (treatments) in rounds 1 and 2. Constrained by the number of *D. radicum* larvae and pupae collected on these plants. Size of the data points reflects the number of *D. radicum* on each plant. **d, e** Conditional predictions of the correlation between functional groups of the above and belowground macroinvertebrates and the abundance of *D. radicum* in round 1 and 2; dots are the original data points, the line indicates the predicted values and the grey area depicts 95% confidence interval. Total *D. radicum* indicates the sum of larvae and pupae. AG: aboveground, BG: belowground

We collected a total of 110 *D. radicum* larvae and 323 pupae from 464 plants in two rounds of sampling. More *D. radicum* larvae / pupae were collected in the second round (Table 1, Fig. 3a), on average 0.96 per plant compared to 0.60 per plant in the first round. Cropping systems affected the number of *D. radicum* larvae / pupae significantly (P = 0.048,Table 1, Fig. 3a), but how cropping systems affected *D. radicum* larval and pupal abundance differed between the rounds (Table 1, Fig. 3a). In the first round, there were no differences between cropping designs. Also in the second round, we found no differences between any of the cropping designs and the reference monoculture. However, we did find more *D. radicum* larvae / pupae on plants grown in strips with wheat combined with broad beans (Strip_additive) compared to plants grown in strips with multiple cultivars of cabbage and wheat (Strip_cultivar).

In both rounds, strip-cropping designs affected cabbage-shoot dry biomass (Table 1, Fig. **S3a**). Plants grown in the reference monoculture had higher biomass than plants in all other cropping designs (Fig. **S3a**). In the first round, there was a strong correlation between plant size and the number of *D. radicum* larvae / pupae (Table 1, Fig. **S3b**), larger plants harboured more cabbage root flies. This correlation was not found in the second round (Table 1, Fig. **S3b**).

### Relation between above- and belowground invertebrate communities and *D. radicum* oviposition and larval / pupal abundance

*D.radicum* oviposition (Fig. 2b, c) and larval / pupal abundance (Fig. 3b, c) were poor predictors of the variation in the aboveground herbivore community and the whole cabbage-invertebrate community, respectively. In the shoot-herbivore communities of plants on which we monitored *D. radicum* oviposition, the strongest contributors to separation between plants in the PCA analyses were *Myzus persicae*, *Pieris rapae* and *Brevicoryne brassicae* in the first round, and *Plutella xylostella* and *Phylotreta undulata* in the second round (Fig. S4). No cabbage shoot herbivore species seemed to correlate with *D. radicum* oviposition in either round, based on PCA loadings (Fig. 2b, c). We further explored the relationship between herbivore functional groups and *D. radicum* oviposition and found no correlation between phloem feeders or leaf chewers and cabbage root fly oviposition (Fig. 2d, e).

Inspection of the strongest contributing factors of the PCA suggests that the number of scarab larvae and earthworms positively correlates with the number of *D. radicum* larvae and pupae in rounds 1 and 2, respectively (Fig. S5b, c). Moreover, the number of wireworms (larvae of click beetles, family Elateridae) is negatively correlated with *D. radicum* larvae / pupae in both rounds in both the PCA and RDA (Fig. 3b, c and Fig. S5b, c). In addition, *B. brassicae* and *P. undulata* contributed to separation in the PCA analyses of round 1 and 2, respectively, but did not correlate with *D. radicum* based on loadings (Fig. S5b, c). We further explored these associations by analysing the correlations between functional groups of above- and belowground macroinvertebrates and the number of *D. radicum* larvae / pupae (Fig. 3d, e). In the first round, no correlation was found between any functional group and *D. radicum* larvae / pupae. However, in the second round, the number of *D. radicum* larvae / pupae negatively correlated with the number of belowground herbivores (Table 1, Fig. 3e). Analyses show that plants with more wireworms, by far the most abundant belowground herbivore other than *D. radicum* (Fig. S5a), harboured fewer *D. radicum* larvae / pupae. Furthermore, there was a positive correlation between the number of *D. radicum* larvae / pupae and belowground predators and detritivores (Table 1, Fig. 3e). There was no correlation between the number of aboveground herbivores, regardless of feeding guild, and *D. radicum* larvae / pupae in either round.

### *Delia radicum* natural enemy communities in different cropping systems

**Fig. 4:**
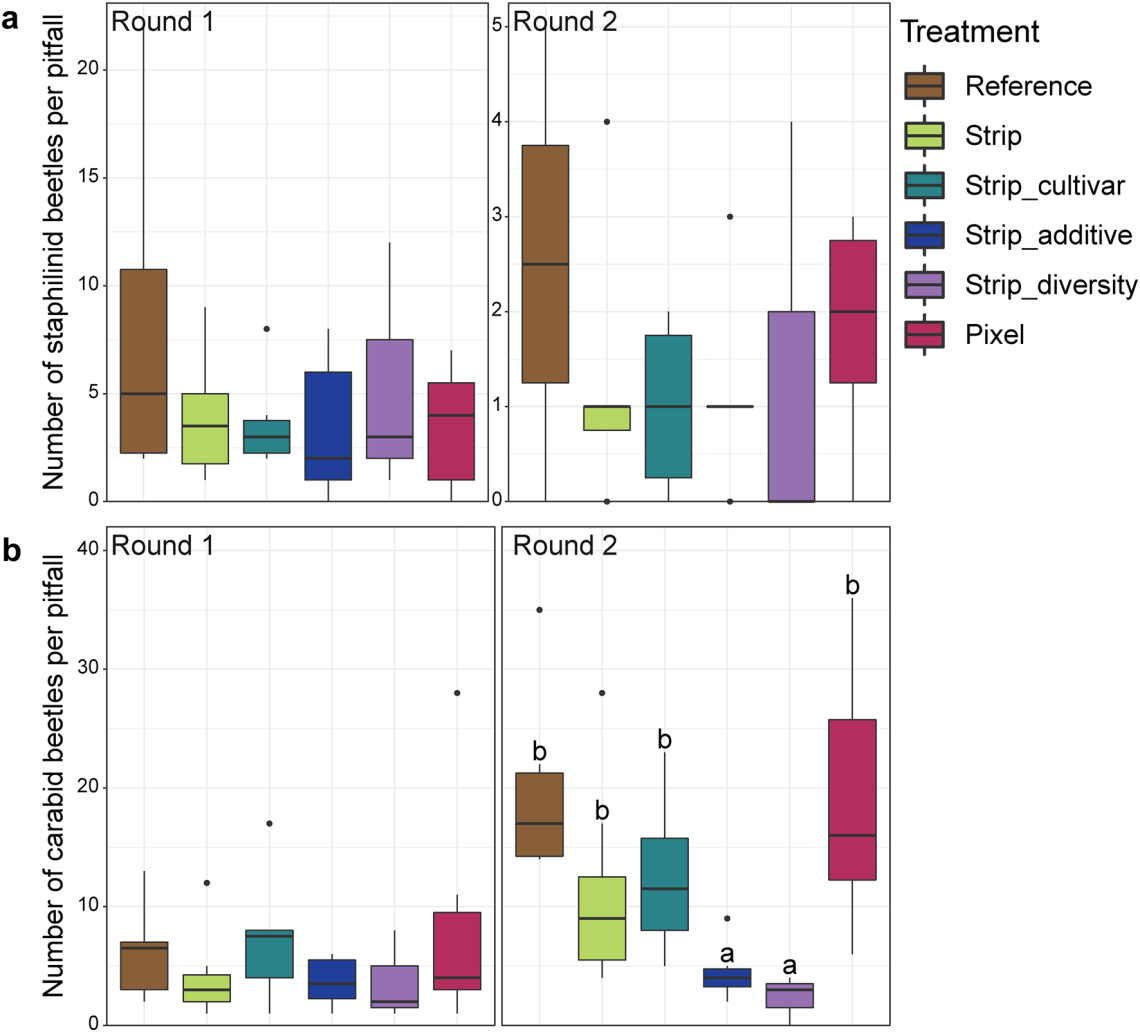
Staphylinid **a** and carabid **b** beetles collected via pitfall trapping in different cropping systems. Results of pairwise comparisons between treatments are indicated with letters; treatments having no letters in common differ significantly (*P* < 0.05)

To assess natural enemies of the cabbage root fly, we collected 209 staphylinid beetles and 627 carabid beetles in two rounds of pitfall trapping. Numbers of beetles per trap were highly variable. The carabid species with the highest activity density were *Pterostichus melanarius* and *Harpalus rufipes* (Fig. **S6**). The number of staphylinid beetles did not differ between the cropping designs (Table 1); more beetles were found in the first than in the second collection round (Table 1; Fig. 4a). Carabid beetles were collected more in the second round (Table 1) and were affected by the cropping designs (Table 1; Fig. 4b). In the second round, we found fewer carabids in the strip design with wheat combined with broad beans (Strip_additive) and the strip design with six crops (Strip_diversity) compared to the other four cropping designs (Reference, Strip, Strip_cultivar and Pixel).

In total, 126 adult insects emerged from the collected fly larvae and pupae: 92 cabbage root flies, 22 *A. bipustulata*, 3 *A. bilineata*, and 9 T*. rapae*. We assume that the majority of flies were *D. radicum*, and however, a small proportion may have been *D. floralis* (Klingen et al. 2000). Most *A. bipustulata* (18) emerged from pupae collected in the first round, whereas most *T. rapae* (8) emerged from pupae collected in the second round. Due to these low numbers, we did not perform statistical analysis on parasitism.

## Discussion

We found that cabbage root fly oviposition was higher in strip-cropping designs, but that this did not result in higher infestation of cabbage plants with larvae and pupae. The highest numbers of cabbage root fly eggs were collected in the most diverse strip-cropping design, which included six main crops (Strip_diversity). Interestingly, the numbers of larvae and pupae per plant did not differ between cropping designs, which might be indicative of a lower survival when plants were grown in more diverse cropping systems. This discrepancy between cabbage root fly oviposition and subsequent larval infestation may have several causes, including abiotic factors, natural enemies, or even plant-mediated interactions with other herbivores. In one of our sampling rounds, we did observe a positive correlation between belowground predators and *D. radicum* larval / pupal abundance, indicating that generalist predators might play some role in control of *D. radicum*. However, carabid and staphylinid beetle activity density was not higher in the more diverse cropping systems. Moreover, the number of collected pupae was too low to draw conclusions on parasitism. Contrary to our expectations based on plant-mediated interactions between above and belowground herbivores (Karssemeijer et al. 2020; Soler et al. 2007), we found no correlation between the number of aboveground phloem feeders or leaf-chewers and the numbers of eggs, larvae or pupae of the cabbage root fly. Other belowground macroinvertebrates collected in our sampling effort did not explain much of the variation in root fly infestation among plants. We found that the number of cabbage root fly larvae and pupae correlated negatively with the abundance of other root herbivores and positively with belowground detritivores, although the effects were weak.

Many ecologists have hypothesized how plant diversity affects insect herbivore communities (Agrawal et al. 2006). The resource concentration hypothesis states that specialist herbivorous insects would find their host plant more easily and stay longer in larger and less diverse host-plant stands (Root 1973). Our results do not support this hypothesis, as we found fewest eggs on plants in the reference monoculture, whereas we found most eggs on plants grown in the most diverse strip cropping system (Strip_diversity). Potentially, cabbages in this cropping design might suffer from associational susceptibility (Agrawal et al. 2006), as the four extra main crop species might supply additional resources that adult *D. radicum* utilize, e.g. pollen or nectar and by offering shelter (Finch and Coaker 1969; Nilsson et al. 2011). For instance, we observed more cabbage root fly eggs on cabbage plants grown in a cropping system that included broad beans as an additional (extra)floral nectar (Strip_additive and Strip_diversity). Here, host-searching behaviour and foraging for food coincide within the cropping system, which may have stimulated oviposition on host plants near the nectar-producing plants. Indeed, this has been recorded in many herbivorous insect species of which the adults feed on floral resources (Wäckers et al. 2007). However, *D. radicum* oviposition was not affected by intercropping cabbage with dill and buckwheat as floral resources (Nilsson et al. 2012). Perhaps broad beans, the main source of (extra)floral nectar in our setup, provide a highly suitable food source for cabbage root flies. On the other hand, plants grown in the pixel-cropping system received few eggs, even though the composition of plant diversity was similar to the Strip_diversity cropping design. Perhaps, the distance between the host crops and the non-host crops in a strip-cropping setting is still sufficiently high to maintain stimulation of oviposition by *D. radicum*. Indeed, a previous study showed that the distance between host cabbage plants and non-host clover plants should be less than 50 cm to result in a reduction of *D. radicum* oviposition (Tukahirwa and Coaker 1982). Alternatively, this finding might be indicative of the neural constraint hypothesis (Agrawal et al. 2006), as non-host plants in close proximity to the cabbages might have interfered with cabbage root fly host-searching behaviour in a similar manner as was demonstrated for a clover undercrop (Finch and Collier 2000; Tukahirwa and Coaker 1982). These results suggest that interference with host-searching behaviour from neighbouring non-host plants is likely low in a strip intercropping system, whereas interference might be a major influence in pixel or mixed cropping systems.

Differences in plant quality could provide an alternative explanation for the higher number of cabbage root fly eggs found in cropping designs with an additional nectar source. The strip cropping designs that included a legume species, e.g. broad bean (Strip_additive and Strip_diversity), also received a plant-based fertilizer instead of farm-yard manure and a precrop with a grass-clover mixture instead of only grass. Thus, plants grown in these strips may differ in nutrient status due to plant-based fertilization and nitrogen-fixing properties of legumes, as compared to manure fertilized strips. Oviposition of cabbage root flies may be affected indirectly by soil nutrients, for instance, increased sulphur content makes oilseed rape plants (*B. rapa*) more attractive (Marazzi and Städler 2005). Moreover, glucosinolate content can be altered in *Brassica* plants grown in pots together with other plant species (Björkman et al. 2008; Ngwene et al. 2017). Such changes in chemical defence may also play a role in our field setup, especially since *D. radicum* females use these glucosinolates as oviposition cues (Hawkes and Coaker 1979; Roessingh et al. 1992). We found a positive correlation between the abundance of all stages of the cabbage root fly and plant size in the first sampling round of our experiments. Laboratory choice experiments have previously shown that *D. radicum* indeed prefers to oviposit on larger plants when given the choice (Koštál and Finch 1994). Interestingly, we did not find this correlation in the later sampling round. Flies earlier in the season may be more sensitive to differences in plant size, or there may be a threshold of plant size that flies use in their oviposition behaviour which most plants had surpassed in the second round. Alternatively, the contrast of plant sizes might be more readily distinguishable early in the season, when soil cover is still relatively low.

Whereas oviposition was positively affected by higher crop diversification, we did not observe an effect of cropping system on the number of larvae and pupae collected. Oviposition assays are used by farmers to assess whether insecticides should be applied (Bligaard et al. 1999; Lamy et al. 2017b). Our results show that this may not be a good proxy for cabbage root fly infestation, especially in diversified cropping systems. Indeed, cabbage yield in the same field trial did not seem to suffer from the high *D. radicum* egg densities: the highest yield was recorded in the Strip_diversity and Strip_additive cropping designs, in which most cabbage root fly eggs were found (Table S2; Ditzler 2022). The discrepancy between collected eggs and later life stages indicates a high mortality of eggs and early instar larvae, especially in those cropping designs in which most eggs were collected.

Many factors could have contributed to mortality of eggs and early instar larvae, including abiotic factors, plant defence and quality, and natural enemies. Eggs and early instars of *D. radicum* can be eaten by carabid and staphylinid beetles (Finch 1989). However, it is difficult to estimate their importance based on the data we collected in this study. Carabid beetles of the genus *Bembidion* are considered important predators of *Delia* species (Björkman et al. 2010; Ferry et al. 2007; Finch and Elliott 1992), but we only collected a very small number of these beetles. The carabid species with the highest activity density in our field, *P. melanarius*, is predominantly carnivorous (Turin 2000). However, the extent to which *P. melanarius* contributes to the control of *D. radicum* eggs and larvae may be low. In a laboratory study, *P. melanarius* did not eat any eggs at all (Finch 1996; Finch and Elliott 1992), although this was contradicted in another experiment (Andersen et al. 1983). While the second most abundant carabid species, *H. rufipes*, does feed on *D. radicum* eggs in vitro, it is considered to be predominantly a seed predator (Andersen et al. 1983; Finch 1996; Turin 2000). We did find a positive correlation between the number of *D. radicum* and soil-dwelling predators in our root–soil samples. This may mean that predators were attracted to infested plants in search of cabbage root fly larvae and pupae to eat, or that similar soil conditions were preferred by both groups. Assays using sentinel *D. radicum* eggs or pupae glued to cards would be a valuable next step to assess predation and parasitism under field conditions (McHugh et al. 2020). Furthermore, future studies should use DNA metabarcoding of the gut contents of potential *D. radicum* predators (e.g. carabids, staphylinids, histerids, ants, centipedes, etc.), as this could identify which arthropod species are actually feeding on *D. radicum* in agricultural fields (Roubinet et al. 2018).

Based on greenhouse experiments on plant-mediated interactions, we expected, the aboveground chewers to positively affect *D. radicum* oviposition, but negatively affect *D. radicum* infestation (Karssemeijer et al. 2020, 2022b; Soler et al. 2007). However, we did not record a significant correlation for either life stage. This suggests that plant-mediated negative effects of aboveground chewers on *D. radicum* found in greenhouse studies are not supported by studies performed in the field (Karssemeijer et al. 2020, 2022b; Soler et al. 2007). A meta-analysis of plant-mediated interactions between above- and belowground herbivores confirms that the effects in fields are much less pronounced than in greenhouse trials (Johnson et al. 2012). The effect sizes in fields might be reduced through multiple-herbivory or legacy effects, as induction soon after planting in a field experiment can affect the herbivore community throughout the growing season (Poelman et al. 2008; Stam et al. 2018, 2019). Since we monitored the aboveground herbivore community only around the same time as our *D. radicum* measurements, we may have missed important earlier inducers of systemic plant defence. We did find a negative correlation between *D. radicum* and other belowground herbivores, mainly consisting of wireworms (Elateridae larvae). In milkweed plants, an asymmetrical interaction was found between specialist root-feeding *Tetraopes tetrophthalmus* larvae and wireworms. The mass of *T. tetrophthalmus* increased when wireworms were present, while the latter decreased in the presence of the other (Erwin et al. 2013). To the best of our knowledge, no studies have specifically investigated interactions between *D. radicum* and other root herbivores when they feed on the same plants.

## Conclusion

For decades, researchers have investigated potential strategies to control *D. radicum*, such as the use of biological control agents (Chen et al. 2003; Finch 1989; Hartfield and Finch 2003; Kapranas et al. 2020), natural plant resistance, the use of cover crops (Finch and Collier 2000; Meyling et al. 2013), and application of push–pull systems (Lamy et al. 2020; Lamy et al. 2017a). With this study, we investigated novel crop diversification methods to counter this devastating insect pest. While we did not find a beneficial effect of strip cropping on cabbage root fly oviposition, our data show that various setups had similar pest pressure, indicative of increased mortality of immature life stages in more diverse cropping systems. Our findings highlight the complexity of plant–insect interactions in the field, in which many factors such as nectar availability and spatial configuration of plants coincide to determine pest dynamics. An integrated approach should be considered to reduce damage in a sustainable manner, combining multiple strategies, including crop diversification, carefully selected crop cultivars, measures to increase natural enemy populations, and novel pest control techniques.

## Author contributions

PK was involved in conceptualization, methodology, formal analysis, investigation, data curation, writing—original draft, writing—review & editing, visualization, and supervision. LC contributed to conceptualization, methodology, investigation, writing—original draft, writing—review & editing, and supervision. Karthick Gajendiran was involved in methodology and investigation. RG contributed to conceptualization, methodology, investigation, and writing—review & editing. DFA contributed to conceptualization, writing—review & editing, supervision, project administration, and funding acquisition. JJAL was involved in conceptualization, writing—review & editing. MD contributed to conceptualization, writing—review & editing, supervision, and funding acquisition. EHP was involved in conceptualization, methodology, writing—review & editing, supervision, project administration, and funding acquisition.

## Supplementary Information

Below is the link to the electronic supplementary material.

Comparison of *D. radicum* egg (**a**, **b**) and larval / pupal (**c**, **d**) abundance among the strip cropping design on each field (a, c) and between the Reference (Ref) and strip cropping designs on the reference field alone (b, d). Results of pairwise comparisons between fields or cropping designs are indicated with letters; fields or cropping designs having no letters in common differ significantly (*P* < 0.05). (PNG 220 KB)

**a** Radius of cabbage plants in different cropping setups, measured as the distance between the furthest leaf tips. Results of pairwise comparisons between treatments are indicated with letters; treatments having no letters in common differ significantly (*P* < 0.05). **b** Correlation between cabbage radius and the number of *D. radicum* eggs collected from those plants in round 1 and 2. NS: Not significant. (PNG 233 KB)

**a** Shoot dry weight of cabbage plants in different cropping setups. Results of pairwise comparisons between treatments are indicated with letters; treatments having no letters in common differ significantly (*P* < 0.05). **b** Correlation between cabbage shoot dry weight and the number of *D. radicum* larvae and pupae collected from those plants in round 1 and 2. NS = Not significant. (PNG 219 KB)

**a** Aboveground herbivore macroinvertebrates on cabbage plants in different cropping designs. Aboveground herbivores on the plants were assessed in the week prior to placement of *D. radicum* egg traps. Different colour schemes indicate different functional groups. **b**, **c** Principal component analyses (PCA) of aboveground macroinvertebrates in round 1 and 2. Size of the data points reflects the number of *D. radicum* eggs recovered in traps placed the subsequent week. (PNG 670 KB)

**a** Above- and belowground macroinvertebrates on and around cabbage plants in different cropping designs. Belowground macroinvertebrates was assessed in a soil core (20 cm diameter, 20 cm depth) around cabbage plants. Aboveground herbivores on the plants were assessed one day prior to taking of soil cores. Different colour schemes indicate different functional groups. **b**, **c** Principal component analyses (PCA) of above- and belowground macroinvertebrates in round 1 and 2. Size of the data points reflects the number of *D. radicum*. (PNG 690 KB)

Pitfall catches of carabids and staphylinids in different cropping designs. Carabid beetles were identified to the species level. (PNG 121 KB)

Supplementary file7 (DOCX 25 KB)

## Data Availability

Data will be made publically available upon acceptance.
