# Supplementary figures and images for: Diverse cropping systems lead to higher larval mortality of the cabbage root fly (Delia radicum)
Source: J Pest Sci (2004). 2023 May 5:1–17. Online ahead of print. doi: 10.1007/s10340-023-01629-1 (PMC10161186; doi:10.1007/s10340-023-01629-1)

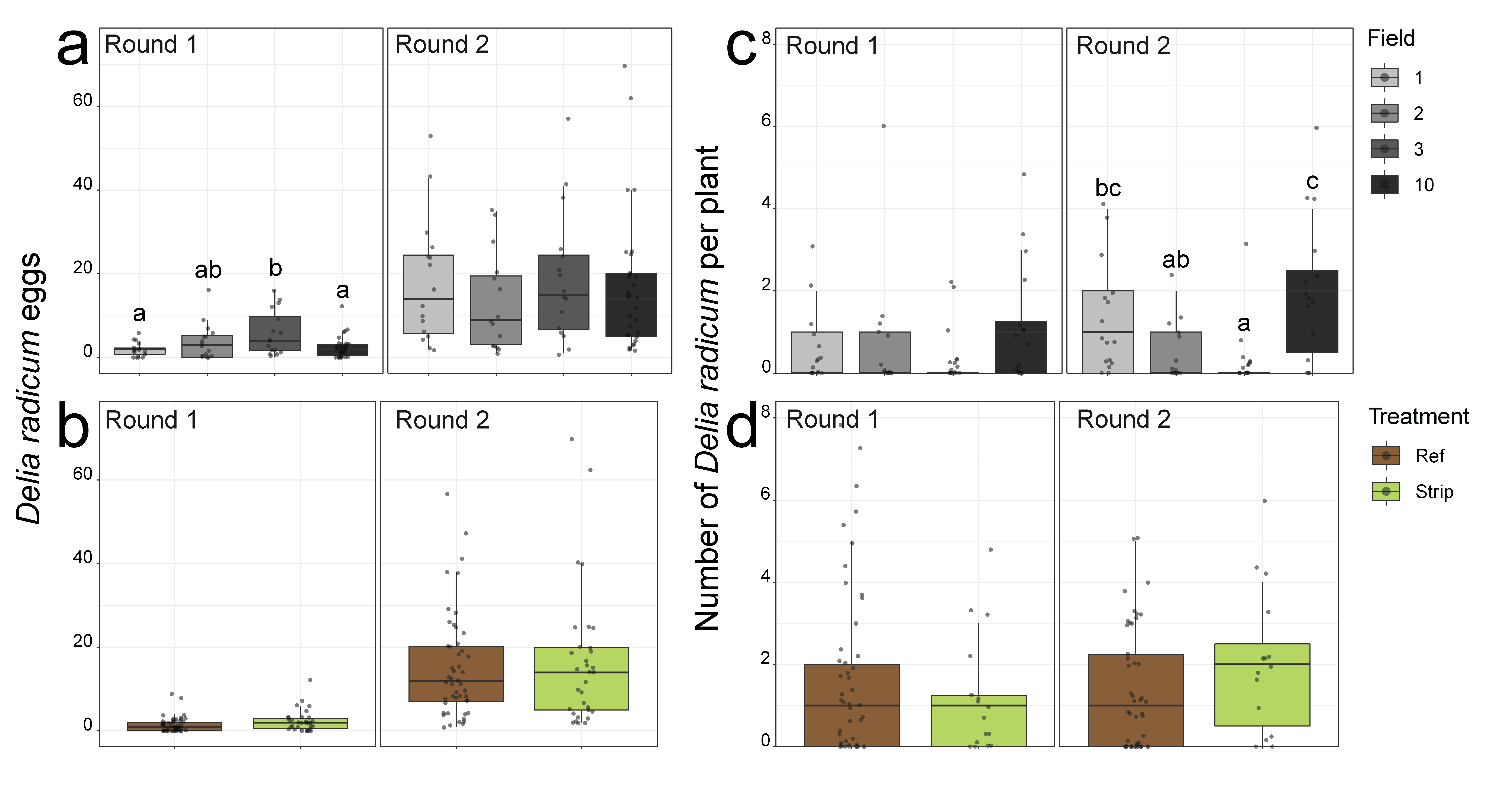

Supplement: Supplementary file 1 — Comparison of D. radicum egg (a, b) and larval / pupal (c, d) abundance among the strip cropping design on each field (a, c) and between the Reference (Ref) and strip cropping designs on the reference field alone (b, d). Results of pairwise comparisons between fields or cropping designs are indicated with letters; fields or cropping designs having no letters in common differ significantly (P < 0.05). (PNG 220 KB) [file 10340_2023_1629_MOESM1_ESM.png]

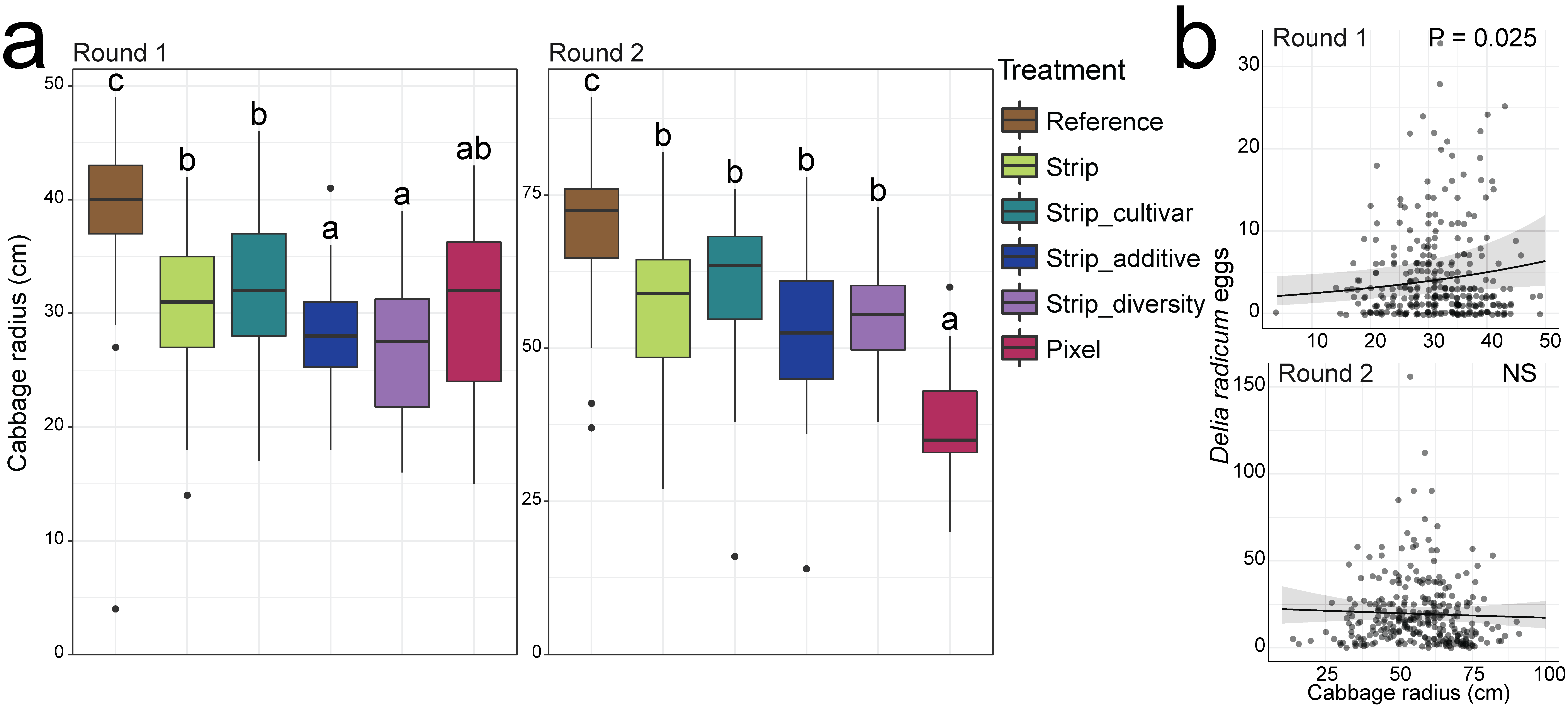

Supplement: Supplementary file 2 — a Radius of cabbage plants in different cropping setups, measured as the distance between the furthest leaf tips. Results of pairwise comparisons between treatments are indicated with letters; treatments having no letters in common differ significantly (P < 0.05). b Correlation between cabbage radius and the number of D. radicum eggs collected from those plants in round 1 and 2. NS: Not significant. (PNG 233 KB) [file 10340_2023_1629_MOESM2_ESM.png]

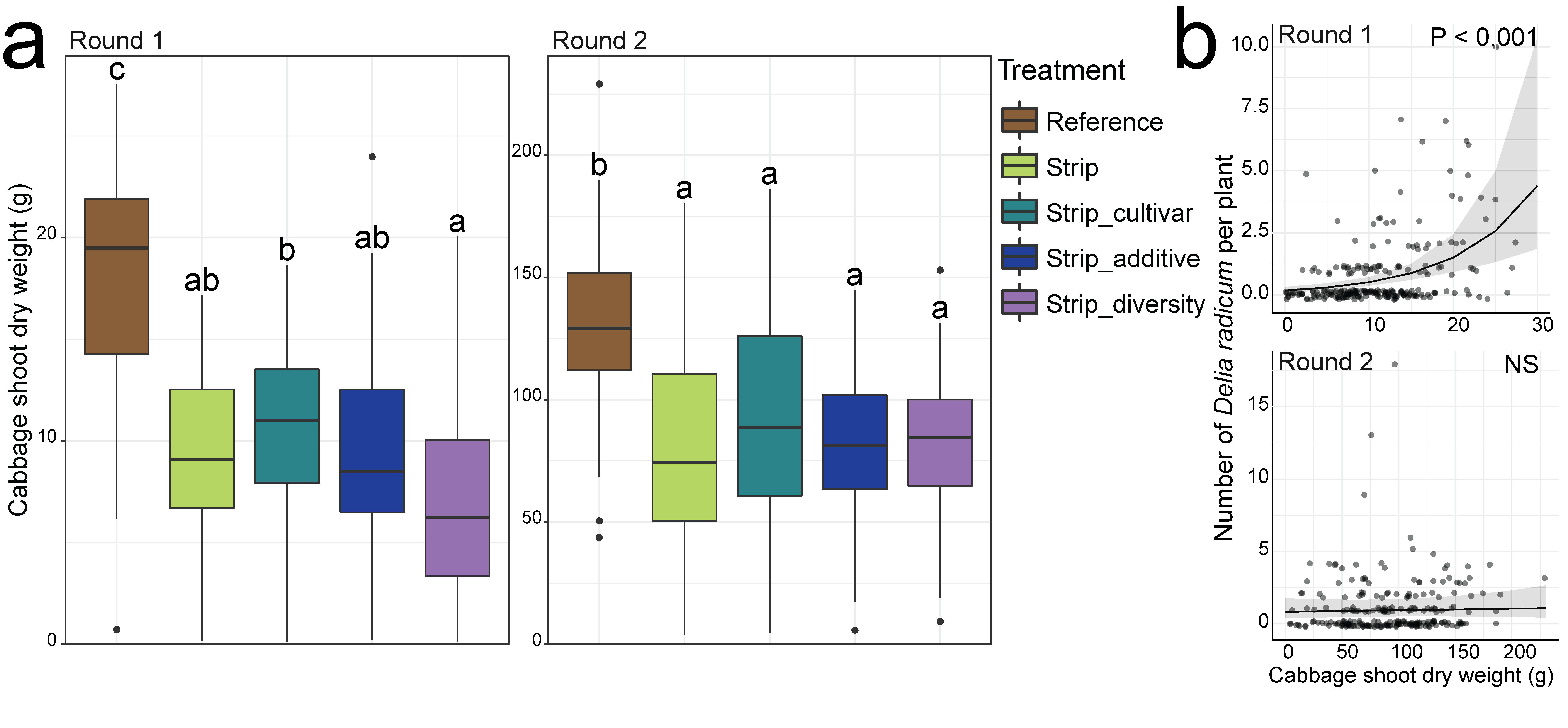

Supplement: Supplementary file 3 — a Shoot dry weight of cabbage plants in different cropping setups. Results of pairwise comparisons between treatments are indicated with letters; treatments having no letters in common differ significantly (P < 0.05). b Correlation between cabbage shoot dry weight and the number of D. radicum larvae and pupae collected from those plants in round 1 and 2. NS = Not significant. (PNG 219 KB) [file 10340_2023_1629_MOESM3_ESM.png]

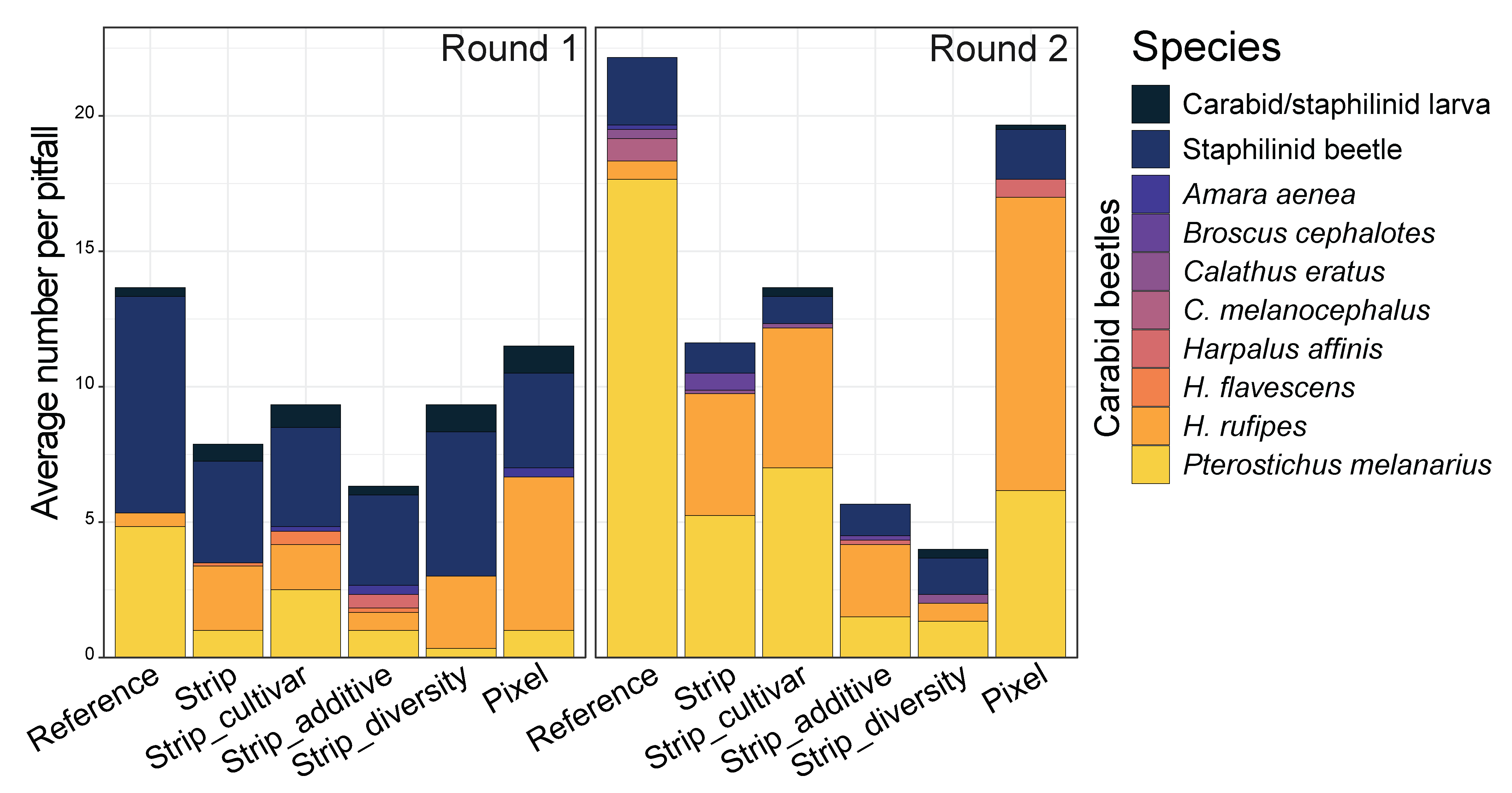

Supplement: Supplementary file 6 — Pitfall catches of carabids and staphylinids in different cropping designs. Carabid beetles were identified to the species level. (PNG 121 KB) [file 10340_2023_1629_MOESM6_ESM.png]
