# Supplementary material for: Diverse cropping systems lead to higher larval mortality of the cabbage root fly (Delia radicum)
Source: J Pest Sci (2004). 2023 May 5:1–17. Online ahead of print. doi: 10.1007/s10340-023-01629-1 (PMC10161186; doi:10.1007/s10340-023-01629-1)
Supplement: Supplementary file 7 — Supplementary file7 (DOCX 25 KB) [file 10340_2023_1629_MOESM7_ESM.docx]

**Table S1.** Number of cabbage root fly larvae and pupae (mean ± standard error) found in a biweekly monitoring effort throughout the field season. Every other week 24 plants were sampled, equally distributed over the four fields. Plants were taken from buffer strips, cabbage strips in between two treatments which are not used for any other measurement, to minimise interference with the field trial. Plants were collected with an augur (20 cm diameter). The roots and soil were carefully assessed for cabbage root fly larvae and pupae.

| **Date** | **N** | **Larvae** | **Pupae** | **Total** |
| --- | --- | --- | --- | --- |
| 15 June 2021 | 23 | 1.17±0.46 | 0.57±0.19 | 1.74±0.49 |
| 29 June 2021 | 24 | 0.83±0.31 | 1.29±0.27 | 2.13±0.50 |
| 13 July 2021 | 24 | 0.50±0.16 | 0.50±0.21 | 1.00±0.31 |
| 27 July 2021 | 24 | 0.96±0.33 | 0.63±0.20 | 1.58±0.45 |
| 10 August 2021 | 24 | 0.38±0.19 | 0.83±0.30 | 1.21±0.42 |
| 24 August 2021 | 24 | 0.04±0.04 | 0.54±0.23 | 0.58±0.22 |
| 7 September 2021 | 24 | 0.00±0.00 | 0.25±0.14 | 0.25±0.14 |
| 21 September 2021 | 24 | 0.00±0.00 | 1.25±0.45 | 1.25±0.45 |
| 5 October 2021 | 24 | 0.00±0.00 | 0.13±0.07 | 0.13±0.07 |

**Table S2.** Cabbage yield (kg/m2) in different cropping systems in 2020. Data courtesy of Dr. Ditzler (Ditzler 2022).

| **Cropping system** | **Mean fresh yield (kg/m2)** | **sd** | **Number of observations** |
| --- | --- | --- | --- |
| Reference | 1.6 | 0.27 | 12 |
| Strip | 1.93 | 0.77 | 32 |
| Strip_cultivar | 1.42 | 0.99 | 24 |
| Strip_additive | 3.11 | 0.68 | 24 |
| Strip_diversity | 2.54 | 0.8 | 12 |
